# Biochemical and Biophysical Characterization of the Deadenylase CrCaf1 from *Chlamydomonas reinhardtii*

**DOI:** 10.1371/journal.pone.0069582

**Published:** 2013-07-23

**Authors:** Jia-Quan Zhang, Guang-Jun He, Yong-Bin Yan

**Affiliations:** State Key Laboratory of Biomembrane and Membrane Biotechnology, School of Life Sciences, Tsinghua University, Beijing, China; Aligarh Muslim University, India

## Abstract

The modulation of mRNA turnover has been increasingly recognized as a hotpoint for gene expression regulation at the post-transcriptional level. In eukaryotic cells, most mRNAs are degraded via the deadenylation-dependent pathway, in which the removal of the poly(A) tail is the initial and rate-limiting step. Caf1, a deadenylase specifically degrades poly(A) from the 3′-end, is highly conserved from yeast to mammalians. Caf1s in higher plants have been shown to be involved in plant development and stress response. However, little is known about the biochemical and biophysical properties of Caf1s in plants. In this research, we cloned the *crcaf1* gene from *Chlamydomonas reinhardtii* and studied the properties of the recombinant proteins. The results showed that CrCaf1 was a deadenylase with conserved sequence motifs, structural features**,** and catalytic properties of the Caf1 family. CrCaf1 degraded poly(A) in a distributive mode with the optimal reacting conditions at pH 7 and 35°C. CrCaf1 had similar activity when coordinated with Mg^2+^ and Mn^2+^, while the enzyme bound to Ca^2+^ or Zn^2+^ was almost inactivated. Zn^2+^ could induce CrCaf1 aggregation with the disruption of the native structure, while Mg^2+^, Mn^2+^ and Ca^2+^ could stabilize CrCaf1 against thermal denaturation by reducing protein aggregation. Among the various metal ions, Mn^2+^ showed the strongest protective effect on CrCaf1 stability, implying that Mn^2+^ might play a role in regulating CrCaf1 stability in the *C. reinhardtii* cells under some stressed conditions. These findings provide a starting point for further investigation of the physiological functions of CrCaf1 in *C. reinhardtii*.

## Introduction

The modulation of mRNA decay is increasingly recognized as an important point for the control of gene expression. In eukaryotic cells, the turnover of mRNAs is regulated by the post-transcriptional modifications at the 5′- and 3′-ends [1], [2]. These non-translational regions help the mature mRNAs to be recognized by various binding proteins, and regulate mRNA processing, transportation, localization, translational efficiency and decay thereby [2]–[4]. Particularly, most mature mRNAs contain a long poly(A) tail at the 3′-end with length up to several hundred nucleotides, which facilitates the circulation of mRNAs during translation and protects mRNAs against degradation by exoribonucleases [4], [5]. The bulk of mRNAs are degraded in a deadenylation-dependent manner, in which the removal of the 3′-end poly(A) tail is the initial and rate-limiting step [1], [6]. Deadenylation is achieved by deadenylases, which are exonucleases that specifically catalyze the hydrolysis of poly(A) from the 3′-end with the release of 5′-AMP as the product. By modulating the length of mRNA poly(A) tail, deadenylases have been found to participate in diverse physiological processes such as embryonic development, DNA damage response, stress response and antiviral response [6]–[11].

CCR4-Caf1, Pan2-Pan3 and poly(A)-specific ribonuclease (PARN) are the best studied members in the deadenylase family [6]. Among them, CCR4 and Caf1 (also known as Pop2 in yeast) are the catalytic components of the CCR4-NOT complex [12], [13], which is a highly conserved mRNA decay machine that has been identified in various organisms ranging from yeast to human beings. CCR4 belongs to the EEP superfamily, while Caf1/Pop2 is a member of the DEDD superfamily [6], [14]. Although CCR4 has been suggested to be the main deadenylase in *Saccharomyces cerevisiae* [13], Caf1/Pop2 is also an active deadenylase *in vitro* and might play an important role in mRNA deadenylation separated from its contact with CCR4 [15], [16]. Although the properties and functions of Caf1 have been well studied in yeast and animals, the physiological roles of CCR4 and Caf1 in plants have not attracted the researchers’ attention until very recently [7], [10], [11]. Sarowar et al. indicated that CaCaf1 is involved in the developmental regulation and pathogen defense of pepper (*Capsicum annuum*) [11]. In *Arabidopsis thaliana*, at least eleven paralogues of yeast Caf1p/Pop2p are found and two homologs (AtCaf1a and AtCaf1b) are proposed to participate in the response of environmental stresses [7], [10], [17]. These observations suggested that Caf1 might have conserved functions such as regulation of growth and development and response to stimuli or stresses in both the animal and plant kingdoms.

Caf1, a DEDDh superfamily exonuclease, possesses the conserved four acidic residues in the active site of the enzymes from most organisms except for *S. cerevisiae*, in which the motif is replaced by SEDQt. Nonetheless, both the yeast and human Caf1 are highly conserved in their tertiary structures [18]–[20]. The four acidic residues DEDD are responsible for the coordination of two divalent metal ions in the active site, which is important for the catalytic activity of Caf1 [21]. PARN, another deadenylase belonging to the DEDDh family, has a high preference for the coordination of Mg^2+^, which is important not only for the activity but also for the stability of the enzyme [22], [23]. However, it seems that Caf1 has no such high preference for one specific divalent metal ion. Human Caf1b/hPop2/CNOT8 has similar catalytic efficiency in the presence of Mg^2+^, Mn^2+^ or Co^2+^ [24], while fission yeast Caf1p/Pop2p prefers Mn^2+^ and Zn^2+^ over Mg^2+^ [25]. Unlike the thoroughly studied yeast and mammalian Caf1s, little is known about the biochemical and biophysical properties of Caf1 from plants. *Chlamydomonas reinhardtii* has long been taken as a model organism in plant sciences including the mRNA processing and stability in plants [26]. Particularly, deadenylation-dependent mRNA decay pathway has also been identified in *C. reinhardtii* [27] although the related deadenylases such as Caf1 and CCR4 have not been characterized yet. In this research, we cloned the *caf1* gene from *C. reinhardtii* and studied the structural and catalytic properties of the enzyme. The results herein provide a starting point for future research of the physiological functions of CrCaf1.

## Materials and Methods

### Materials

Ex Taq DNA polymerase, Pfu DNA polymerase, restriction endonucleases and DNA ligase, 20-mer oligo(A) (A20) were obtained from Takara biotechnology Co., Ltd (Dalian, China). Isopropyl-1-thio-β-D-galactopyranoside (IPTG), imidazole, Tris, AMP, dithiothreitol (DTT) were purchased from Promega. All other reagents were local products of analytical grade.

### Cloning of the *crcaf1* Gene

The total cDNA of the *Clamydomonas reinhardtii* cells was kindly provided by Professor Junmin Pan (Tsinghua University, China). The coding sequence of CrCaf1 (GenBank Accession No. **NW_001843754**) was cloned by PCR using the following primers: sense-primer1 (5′-ACCCGCTTAGCCCAGAACA-3′), antisense-primer1 (5′-CACGGCACCCAGAAACCTTA-3′), sense-primer2 (5′-GGGAATTCCATATGATGAGTCAACTGGCGAGCCTGG-3′), antisense-primer2 (5′-CCGGAATTCTCACGAGCCGTTGTCCTGGC-3′). After digestion of the PCR product with *Nde*I and *EcoR*I, the gene was cloned to the vector pET-28a (Novagen) and verified by sequencing.

### Protein Expression, Purification and Sample Preparation

The recombinant proteins were overexpressed in *Escherichia coli* BL21 (DE3) (Stratagene, Heidelberg, Germany) and purified using a procedure similar to human Caf1b/hPop2 with some modifications [24]. In brief, the recombinant strain was inoculated in 5 ml LB medium with the addition of 50 µg/ml kanamycin and grown overnight at 37°C. The cultures were diluted (1∶100) in LB medium and cultivated at 37°C to reach an OD_600_ value of 0.6. To optimize the conditions of the recombinant protein expression, the incubation temperature and IPTG concentration were screened. The optimal conditions for overexpression are cultivation at 16°C for 20 h with a final IPTG concentration of 0.4 mM. After overexpression, the recombinant strains were lysed by sonication in Lysis Buffer (20 mM Tris-HCl, 500 mM KCl, 20 mM imidazole, 20%(v/v) glycerol,pH8.0). The cell extracts was separated by centrifugation and the supernatant was filtrated twice with 0.22 µm membrane. The His-tagged CrCAF1 proteins were purified by metal-chelated affinity chromatography using a 5 ml HisTrap HP column (GE Healthcare) with 35 mM imidazole in the washing buffer and 250 mM imidazole in the elution buffer. The purified final products with homogenous oligomeric states were collected by size exclusion chromatography (SEC) using a Superdex G-200 column equipped on an ÄKTA purifier (GE Healthcare). The purity of the final products was above 95% as estimated by SDS-PAGE and SEC analysis. The protein concentration was determined according to the Bradford method [28]. The protein samples were prepared in buffer A containing 20 mM Tris-HCl (pH 8.0), 100 mM KCl, 0.5 mM DTT, 0.2 mM EDTA and 20% (v/v) glycerol.

### Spectroscopy

The spectroscopic experiments were determined using protein samples in buffer A with a final concentration of 0.20 mg/ml (6 µM). Far-UV circular dichroism (CD) spectra were performed on a Jasco-715 spectrophotometer (Jasco, Tokyo, Japan) using a cell with a path length of 0.1 cm. Intrinsic fluorescence spectra were measured on a Hitachi F-2500 spectrophotometer (Hitachi, Tokyo, Japan) using a 0.2 ml cuvette with an excitation wavelength of 295 nm. All spectroscopic experiments were repeated at least three times. The fitting of the fluorescence spectra was performed using the discrete states model of Trp residues in proteins [29], [30] and was calculated by a program developed in-house based on the SIMS algorithms of decomposition [31]. Raleigh resonance light scattering (RRS) was recorded on a Hitachi F-2500 spectrophotometer at 90° with an excitation wavelength of 295 nm as described elsewhere [32].

### Homology Modeling

The automated protein structure homology modeling was performed using SWISS-MODEL [33]. The crystal structure of human Caf1a (PDB ID: 2D5R, chian A) [20] was used as the template structure. The structures were manipulated and rendered using PyMol (http://www.pymol.org/).

### Enzyme Assay

The enzymatic activity was measured according to the SEC method as described previously [34]. In brief, the standard reaction buffer contained 20 mM Tris–HCl, pH 7.0, 100 mM KCl, 1.5 mM, MgCl_2_, 0.5 mM DTT, 0.2 mM EDTA and 10% (v/v) glycerol. SEC experiments were performed on an ÄKTA purifier equipped with a Superdex 200 10/30 GL column (GE Healthcare). The reaction was initiated by mixing 20 µl enzyme and 100 µl substrate stock solutions and the final concentration of CrCaf1 was 0.05 mg/ml. After incubated for a given time, the samples were cooled on ice to quench the reaction. Then the samples were loaded on the injection ring with the volume of 100 µl as soon as possible. The absorbance at 280 nm, 254 nm and 215 nm were monitored simultaneously. The concentration of the product AMP was evaluated by the peak area of AMP in the elution profile using the standard curve determined using commercial AMP. All the materials used in the activity assay were RNase-free, and all the experiments were performed in clean bench and ice. The enzymatic kinetic constants *K*_m_ and *V*_max_ were obtained by fitting the data using the Michaelis–Menten equation. *k*_cat_ was calculated by dividing *V*_max_ by the enzyme concentration.

### Thermal Denaturation and Aggregation

The samples were heated continuously as the temperature increased from 25°C to 65°C with an interval of 2.5°C. The temperature was controlled by a circulating water bath. At each given temperature, the samples were equilibrated for 3 min. The thermal denaturation of CrCaf1 was monitored by recording the fluorescence emission spectrum on a Hitachi F-2500 spectrophotometer using a 1 ml cuvette with an excitation wavelength of 295 nm. The thermal aggregation of the samples was monitored by measuring the turbidity at 400 nm (*A*_400_) with an Ultraspec 4300 pro UV/Visible spectrophotometer using a 1 ml cuvette. The aggregation kinetics was obtained by heating the protein samples at a given temperature, and the turbidity at 400 nm was recorded every 2 s. The kinetic data were analyzed along a first-order aggregation reaction using the following equation 35,36:
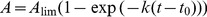
(1)where *t* is the time of incubation at a given temperature, *A*_lim_ is the *A*_400_ value at the infinite time and *k* is the rate constant of the first order reaction. Data fitting was performed by nonlinear regression analysis using the software Prism (GraphPad Inc.).

### Ion Binding Affinity by Fluorescence Quenching

Quantitative evaluation of the binding constants between proteins and the divalent metal ions was determined by the fluorescence quenching method. The fluorescence spectra were measured on a Hitachi F-4500 spectrophotometer at 25°C with an excitation wavelength of 295 nm. The sample volume of the titration experiments was 400 µl. Aliquots of 1 µl stock solutions were added to the samples and equilibrated for 5 min. Then the fluorescence emission spectra were recorded. The change in the fluorescence intensities at 329 nm was used for the calculation of the apparent dissociation constant (*K*_d_) according to the following equation,
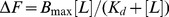
(2)where Δ*F* is the change in the fluorescence intensity induced by the ligand, *B*_max_ is the coefficient, [L] is the concentrations of ions. Data fitting was performed by nonlinear regression analysis using the software Prism (GraphPad Inc.).

## Results and Discussion

### CrCaf1 Contains the Characteristic Motifs of the DEDDh Family

**Figure 1:**
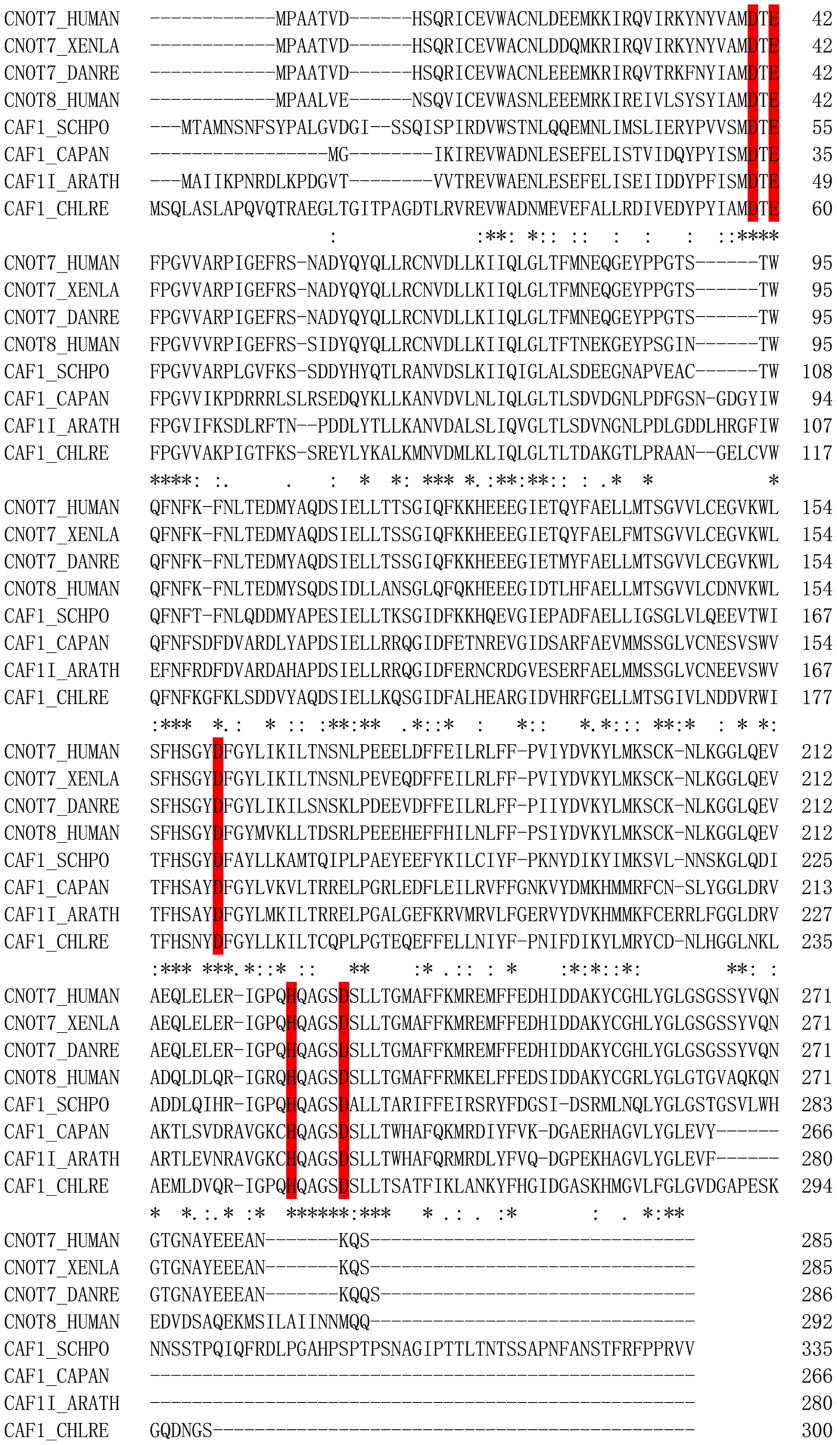
Sequence alignment of Caf1s. The sequences used for the alignment are: human Caf1a/CNOT7 (NP_037486), *Xenopus laevis* Caf1a/CNOT7 (NP_001089689), *Danio rerio* Caf1a/CNOT7 (NP_001070723), human Caf1b/hPop2/CNOT7 (NP_004770), *Schizosaccharomyces pombe* Caf1p/Pop2p (NP_588385), *Capsicum annuum* Caf1 (DQ672569), *Arabidopsis thaliana* Caf1I (NP_190012) and *Chlamydomonas reinhardtii* (XP_001697447). The characteristic residues of the DEDDh family exonucleases are highlighted by red background.

**Figure 2:**
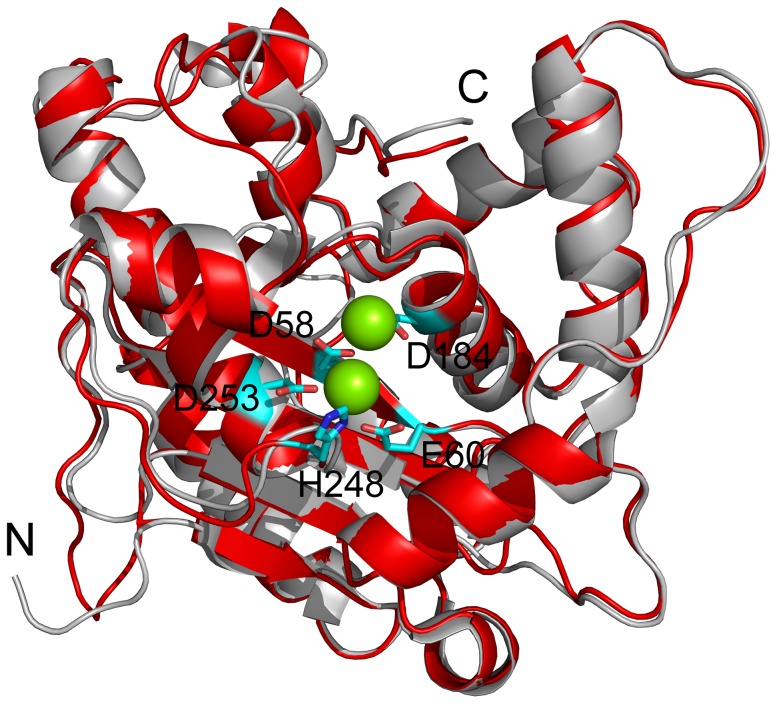
A comparison of the crystal structure of *S. pombe* Caf1p/Pop2p (gray, PDB ID: 2P51) and the predicted structure of CrCaf1 (red). The homology modeling of CrCaf1 structure was performed by SWISS-MODEL using the crystal structure of human Caf1a/CNOT7 (PDB ID: 2D5R) as the template structure. N and C are the N- and C-termini of the proteins. The positions of the four acidic residues and an additional His in the active site are highlighted by stick representation. The two Mg^2+^ in the crystal structure of Caf1p are also presented by space-filling representation.

There is only one yeast Caf1p/Pop2p homolog in *Chlamydomonas reinhardtii*, which is named CrCaf1 in this research. To speculate whether CrCaf1 is a potential deadenylase, sequence alignment was performed among Caf1s from various model organisms. As shown in Figure 1, CrCaf1 shares a high homology with the other well-characterized Caf1s, and has a sequence identity of ∼60% to hCaf1a/CNOT7, hCaf1b/CNOT8 or AtCaf1a and 55% to Caf1p/Pop2p from *S. pombe*. More importantly, the consensus motif of the DEDDh sub-family in the RNase D exonucleases, which includes four acidic residues and an additional His residue, is fully conserved in CrCaf1. The high homology between CrCaf1 and human Caf1a/CNOT7 makes it possible to construct a predicted structure of CrCaf1 by homology modeling using the crystal structure of hCaf1a [20] as the template. The modeled CrCaf1 structure was similar to that of hCaf1a with almost superimposed core structures and some variations in the loops (data not shown). The modeled structure of CrCaf1 also showed a high similarity to that of yeast Caf1p/Pop2p (Figure 2) although the crystal structure of *S. pombe* Caf1p [19] was not used as a template for homology modeling. A high conservation was observed for the structure of the active site. The two divalent metal ions in the active site of yeast Caf1p/Pop2p were well-defined by the side chains of Asp58, Glu60, Asp184, Asp253 and His248 in CrCaf1, suggesting that CrCaf1 was very likely to maintain the catalytic activity of the Caf1 deadenylase family.

### Biophysical Characterization of the Recombinant CrCaf1

**Figure 3:**
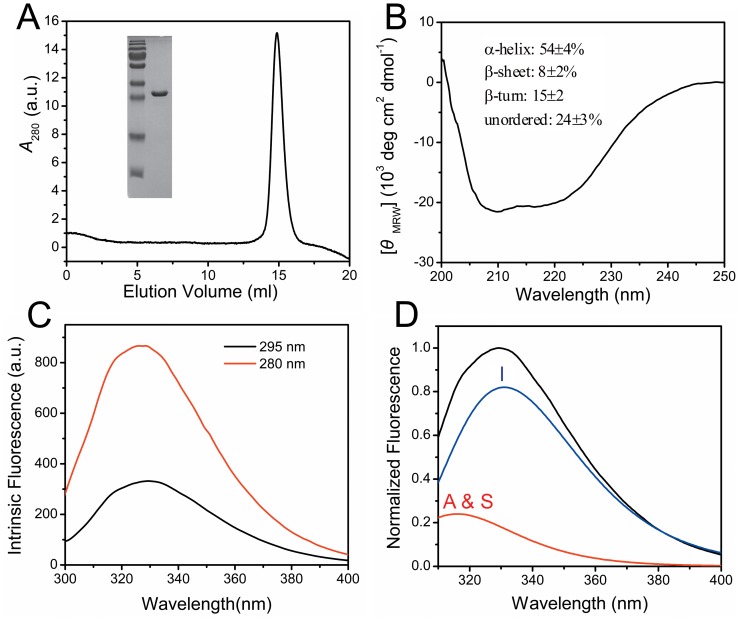
Biophysical characterization of the recombinant CrCaf1. (A) SEC analysis. The inset shows the identification of the recombinant proteins by SDS-PAGE. (B) CD spectrum and the percentages of various secondary structure components. (C) Intrinsic fluorescence of CrCaf1 with an excitation wavelength of 280 nm or 295 nm. (D) Fitting of the Trp fluorescence spectrum obtained by exciting at 295 nm by the theoretical model of discrete states of Trp fluorophores in proteins. The samples for the biophysical experiments were prepared in buffer A with a final concentration of 0.2 mg/ml.

The full-length CrCaf1 coding sequence cloned from *C. reinhardtii* cDNA was subcloned into the pET-28a plasmid for the production of recombinant proteins. The N-terminal His-tagged CrCaf1 was overexpressed in *E. coli* BL21 (DE3), and the soluble fractions were subjected to further purification by Ni^2+^-affinity chromatography and SEC sequentially. The production yield of the soluble recombinant proteins was 10–20 mg/L. The oligomeric status of the purified proteins was characterized by SEC analysis. As shown in Figure 3A, the elution volume of CrCaf1 in the SEC profile using Superdex 200 10/30 GL column was about 14.9 ml. This indicated that the recombinant CrCaf1 existed as a monomer in solutions, which was similar to Caf1s from the other species [18], [19], [24], [37].

The structural features of the CrCaf1 were investigated by spectroscopic methods. The obvious two negative peaks at 208 nm and 222 nm in the far-UV CD spectrum (Figure 3B) of CrCaf1 revealed that the secondary structures of CrCaf1 had a high content of helical structures. This deduction was further supported by prediction of the percentages of secondary structure components using CDPro [38]. The result from CD spectroscopy is consistent with that from homology modeling (Figure 2). The tertiary structure of CrCaf1 was assessed by intrinsic fluorescence (Figure 3C), which could reflect the microenvironments around the aromatic residues. When excited at 280 nm, both Trp and Tyr fluorescence can be observed, while only the Trp fluorophore is excited using an excitation wavelength of 295 nm. The emission maximum wavelength was ∼229 nm for both the fluorescence spectra excited at 280 nm or 295 nm, which is similar to that of human Caf1a/CNOT7 [37] or Caf1b/hPop2/CNOT8 [24]. To confirm the result from homology modeling, the Trp fluorescence spectrum obtained by exciting at 295 nm was further analyzed using the theoretical model of discrete states of Trp fluorophores in proteins [29], [30]. The fitting of the fluorescence spectrum suggested that CrCaf1 contained about 20% Class A & S and 80% Class I fluorophores, which correspond to Trp fluorophores in highly hydrophobic microenvironment or interior of the molecule without accessibility to solvents, respectively [29]. There are three Trp residues in CrCaf1 (Trp34, Trp117 and Trp176), all of which were buried in the interior of the modeled structure. Moreover, the side chains of Trp residues in Crcaf1 were almost superimposed with those from human Caf1a or yeast Caf1p (data not shown). These spectroscopic observations confirmed the homology modeling result and indicated that CrCaf1 possessed a typical structure of the Caf1 deadenylase family.

### Enzymatic Activity of CrCaf1

**Figure 4:**
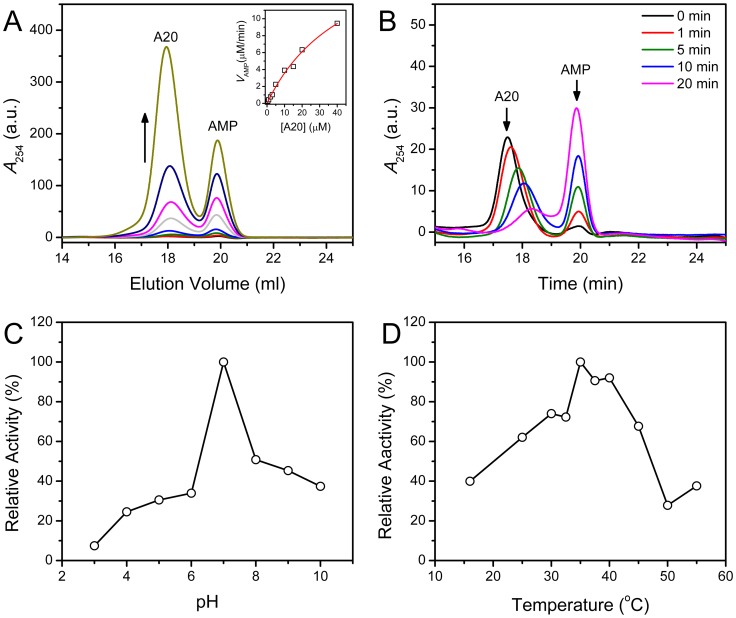
Enzymatic properties of CrCaf1. (A) Determination of the enzymatic parameters by varying the concentration of the substrate A20. The concentration of A20 ranged from 0 to 40 µM, and the concentration of CrCaf1 was 0.05 mg/ml. The reaction time was 10 min. The arrow indicates the direction of increasing the substrate concentration. The inset shows the data fitting using the Michaelis–Menten equation, and the fitted curve is presented as a solid line. (B) Catalytic pattern of CrCaf1 by the SEC method. The SEC profiles were collected at various time intervals. The positions of the substrate A20 and the product AMP are labeled. (C) Characterization of the optimal pH for CrCaf1 reaction. (D) Temperature-dependence of CrCaf1 activity.

The enzymatic activity of CrCaf1 was evaluated by either the methylene blue assay [39] or the SEC method [34]. The results from the methelene blue assay had very large errors, which might be caused by the low activity of CrCaf1. Thus the SEC method was applied to characterize the enzymatic properties of CrCaf1 by using A20 (20-mer oligonucleotide) as the substrate. The enzymatic parameters were obtained by varying the concentration of the substrate A20 (Figure 4A). The data were fitted to the Michaelis-Menten equation (inset of Figure 4A), which yielded the *K*_m_ value of 48±9 µM and the *V*_max_ value of 21±3 µM/min. The *k*_cat_ value was calculated to be 0.25 s^−1^. Compared to the highly active deadenylase PARN [34], the *K*_m_ value of CrCaf1 was about 200-fold larger, while the *k*_cat_ value was about 35-fold smaller than those of PARN. Since no enzymatic parameters of the other Caf1s were obtained using A20 as the substrate, it is difficult to compare the enzymatic properties among Caf1s. However, it has been shown that the relative activity of human Caf1b/hPop2/CNOT8 was less than 1% of that of PARN when using commercial poly(A) as the substrate [24], suggesting that various Caf1s might possess rather low catalytic efficiency when compared with PARN. Furthermore, the large discrepancy in the efficiency of degrading poly(A) between Caf1 and PARN implied that the various deadenylases might participate into dissimilar physiological processes with different requirements of poly(A) removal efficiency. The catalytic pattern of CrCaf1 was studied using the SEC method. As shown in Figure 4B, the amount of the product AMP increased linearly as a function of reaction time, while the peak of the substrate moved continuously towards larger elution volumes corresponding to low-molecular-weight molecules. According to the criteria proposed previously [34], these observations implied that CrCaf1 degraded A20 in a highly distributive mode. The pH and temperature titration experiments showed that, similar to human Caf1b/hPop2 [24], the optimum conditions for the catalytic activity of CrCaf1 were at pH 7.0 and 35°C (Figures 4C and 4D).

### Effects of Divalent Metal Ions on CrCaf1 Activity and Stability

**Figure 5:**
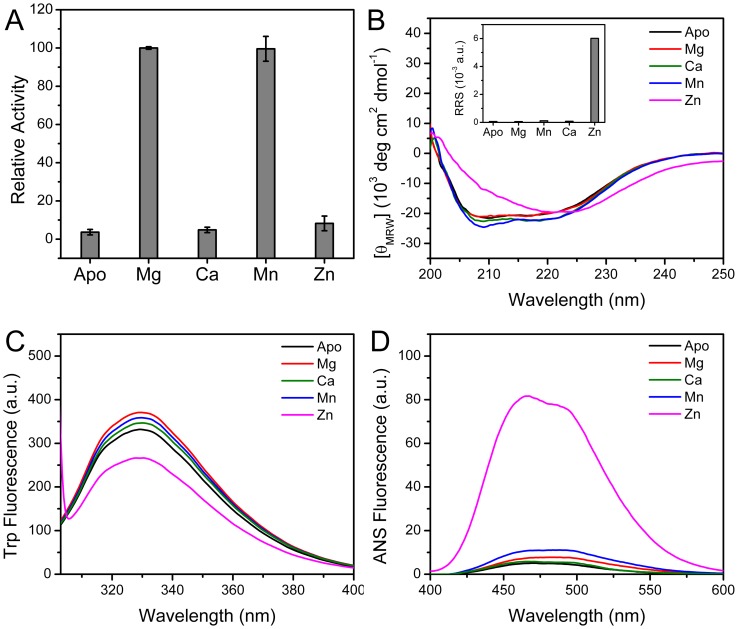
Effects of the divalent metal ions on CrCaf1 activity and structure. (A) Effect of 3 mM Mg^2+^, Ca^2+^, Mn^2+^ and Zn^2+^ on CrCaf1 activity. The experimental conditions of the SEC assay were the same as those described in Figure 4. (B–D) Spectroscopic determination of the effect of 3 mM Mg^2+^, Ca^2+^, Mn^2+^ and Zn^2+^ on CrCaf1 structure by CD (B), Trp fluorescence (C) and ANS fluorescence (D). The inset of panel (B) shows the Raleigh resonance light scattering (RRS), a sensitive tool to probe protein aggregation, of the samples containing various divalent metal ions. The experimental conditions of the spectroscopic experiments were the same as those described in Figure 3.

CrCaf1 belongs to the DEDD superfamily, which is expected to coordinate two divalent metal ions in the active site (Figure 2). To characterize the metal ion preference of Crcaf1, apo-enzyme was prepared by purifying the recombinant proteins with buffer containing EDTA. The apo-enzyme was inactive (Figure 5A), confirming that the coordination of metal ions in the active site is indispensible for CrCaf1 catalytic activity. A screen of various divalent metal ions indicated that CrCaf1 was almost inactive in the presence of 3 mM Ca^2+^ and Zn^2+^, while exhibited similar catalytic activity towards A20 in the presence of 3 mM Mg^2+^ and Mn^2+^. It is interesting that most characterized Caf1s including CrCaf1 had a similar preference to Mg^2+^ and Mn^2+^ [19], [24], [25], which is quite different from the highly Mg^2+^-dependent catalysis of PARN [22].

Spectroscopic techniques were applied to gain insight into the mechanism underlying the dissimilar behavior of various metal ions. As shown in Figure 5B, the coordination of Mg^2+^, Ca^2+^ and Mn^2+^ did not significantly affect the ellipticity and shape of the CD spectra. However, the CD spectrum of CrCaf1 was dominated by β-sheet structures in the presence of Zn^2+^ as revealed by the single negative peak centered at around 220 nm. We further showed that the disruption of the native secondary structure by Zn^2+^ was caused by protein aggregation as reflected by the extremely high light scattering (inset of Figure 5B). Coincident with the increase in light scattering, the ANS fluorescence of CrCaf1 was dramatically increased by Zn^2+^ when compared to the other metal ions (Figure 5D), indicating that a large amount of hydrophobic exposure was induced by the addition of Zn^2+^. The Trp fluorescence of CrCaf1 was slightly increased with the addition of Mg^2+^, Ca^2+^ and Mn^2+^, indicating that the fluorescence intensity change could be used as a sensitive probe to determine the binding affinities between the enzyme and the ions. The titration data (data not shown) indicated that the apparent dissociation constants (*K*_d_) for Mg^2+^ (1.6±0.3 µM) and Mn^2+^ (1.0±0.2 µM) were similar, while the binding of Ca^2+^ was much weaker with *K*_d_>50 µM.

**Figure 6:**
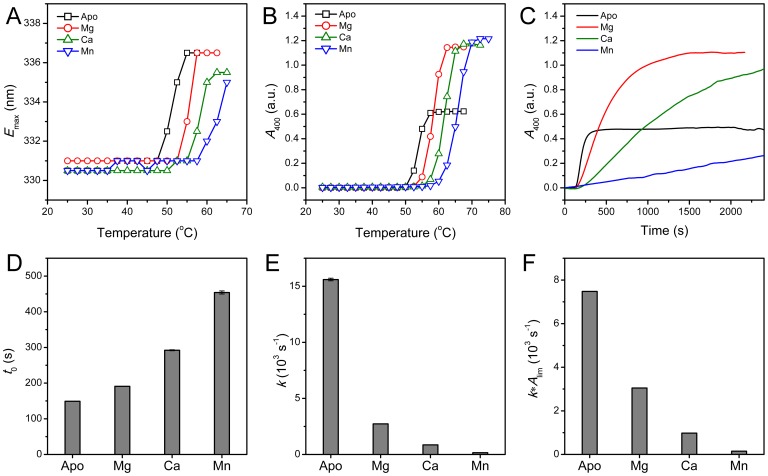
Effect of the divalent metal ions on CrCaf1 thermal stability. (A) Thermal denaturation of CrCaf1 in the presence or absence of divalent metal ions probed by the maximum emission wavelength (*E*_max_) of the intrinsic Trp fluorescence. (B) Thermal aggregation of CrCaf1 monitored by turbidity (absorbance at 400 nm). (C) Effect of 3 mM divalent metal ions on the thermal aggregation kinetics of CrCaf1. The time-course change of the turbidity was recorded by heating the protein samples at 55°C controlled by a water-bath. The data were fitted to Eq. (1), and the kinetic parameters are presented in panels D–F. (D) The lag time *t*_0_. (E) The aggregation rate constant *k*. (F) The initial velocity of aggregation *k*∗*A*_lim_. The concentration of the protein was 0.2 mg/ml, and that of the divalent metal ions was 3 mM.

Previous results have shown that the coordination of Mg^2+^ stabilizes the active site of PARN, but promotes the aggregation of the 54 kDa PARN isoform at high temperatures [23], [36]. The effects of divalent metal ions on CrCaf1 stability was studied by thermal denaturation experiments. As shown in Figure 6, the presence of divalent metal ions could significantly stabilize CrCaf1 by increasing the midpoint temperature of denaturation (Figure 6A) and aggregation (Figure 6B). Among the three metal ions studied herein, Mn^2+^ was the strongest stabilizer, followed by Ca^2+^, while Mg^2+^ was the weakest. It is interesting that Ca^2+^ could also protect CrCaf1 against denaturation although the enzyme was inactive in the presence of Ca^2+^. This suggested that the coordination of divalent metal ions in the active site might modulate the repulsion of the negative charges of the four acidic residues, which further stabilized the protein. It is worth noting that the turbidity, which reflects the size and amount of the aggregates, is not a good evaluator of the action of cosolutes in some cases [35], [36]. Thus a quantitative evaluation was achieved by measuring the aggregation kinetics at 55°C (Figure 6C). The kinetic data were fitted to Eq. (1), and the parameters were shown in Figures 6D–6F. These parameters clearly indicated that the divalent metal ions were stabilizers by elongating the lag time *t*_0_, slowing the rate constant *k* and decreasing the initial velocity k*A_lim_.

In summary, we have cloned the *crcaf1* gene from the *C. reinhardtii* total cDNAs and studied the enzymatic properties and biophysical characteristics of CrCaf1. The results showed that CrCaf1 was a deadenylase with conserved structural features and catalytic properties of the Caf1 family. *C. reinhardtii* has been widely used to address the origin of gene functions in both animal and plant kingdom. Considering that the deadenylation-dependent mRNA decay pathway has been identified in *C. reinhardtii* [27], our findings provide a starting point to further study the physiological functions of CrCaf1 in *C. reinhardtii*. Moreover, we showed that CrCaf1 had similar preference to Mg^2+^ and Mn^2+^ during catalysis, while the enzyme bound to Ca^2+^ and Zn^2+^ in the active site had very low activity. The binding with Zn^2+^ resulted in CrCaf1 aggregation with the disruption of the native structure. Interestingly, both the metal ions essential (Mg^2+^ and Mn^2+^) and nonessential (Ca^2+^) for catalysis could successfully stabilize CrCaf1 against thermal denaturation by reducing the occurrence of protein aggregation. Among the three metal ions, the enzyme coordinated with Mn^2+^ had the highest catalytic activity and stability. Considering that the intracellular free Mg^2+^ concentration was the highest among various divalent metal ions, it is more likely that CrCaf1 coordinated Mg^2+^ in the *C. reinhardtii* cells under normal conditions. However, the strong stabilization effect of Mn^2+^ suggested that Mn^2+^ might play a regulatory role for the cells under abnormal or stressed conditions. Further research is needed to verify whether Mn^2+^ play a role in the regulation of CrCaf1 stability and function *in vivo*.
